# Detection of *NTRK* fusions by RNA-based nCounter is a feasible diagnostic methodology in a real-world scenario for non-small cell lung cancer assessment

**DOI:** 10.1038/s41598-023-48613-4

**Published:** 2023-12-01

**Authors:** Rodrigo de Oliveira Cavagna, Edilene Santos de Andrade, Monise Tadin Reis, Flávia Escremim de Paula, Gustavo Noriz Berardinelli, Murilo Bonatelli, Gustavo Ramos Teixeira, Beatriz Garbe Zaniolo, Josiane Mourão Dias, Flávio Augusto Ferreira da Silva, Carlos Eduardo Baston Silva, Marina Xavier Reis, Erika Lopes Maia, Thainara Santos de Alencar, Alexandre Arthur Jacinto, Rachid Eduardo Noleto da Nóbrega Oliveira, Miguel A. Molina-Vila, Letícia Ferro Leal, Rui Manuel Reis

**Affiliations:** 1 Molecular Oncology Research Center, Barretos Cancer Hospital, 1331, Antenor Duarte Villela, Barretos, São Paulo 14784-400 Brazil; 2 Molecular Diagnostic Laboratory, Barretos Cancer Hospital, Barretos, Brazil; 3 Department of Pathology, Barretos Cancer Hospital, Barretos, Brazil; 4 Barretos School of Health Sciences Dr. Paulo Prata–FACISB, Barretos, Brazil; 5 Department of Medical Oncology, Barretos Cancer Hospital, Barretos, Brazil; 6 Deparment of Radiology, Barretos Cancer Hospital, Barretos, Brazil; 7 Deparment of Thoracic Surgery, Barretos Cancer Hospital, Barretos, Brazil; 8 Laboratory of Oncology/Pangaea Oncology, Dexeus University Hospital, Barcelona, Spain; 9 Life and Health Sciences Research Institute (ICVS), School of Medicine, University of Minho, Braga, Portugal; 10 ICVS/3B’s–PT Government Associate Laboratory, Braga/Guimarães, Portugal

**Keywords:** Non-small-cell lung cancer, Genetic markers, Cancer, Cancer genetics, Lung cancer, Tumour biomarkers, Diagnostic markers

## Abstract

*NTRK1, 2,* and *3* fusions are important therapeutic targets for NSCLC patients, but their prevalence in South American admixed populations needs to be better explored. *NTRK* fusion detection in small biopsies is a challenge, and distinct methodologies are used, such as RNA-based next-generation sequencing (NGS), immunohistochemistry, and RNA-based nCounter. This study aimed to evaluate the frequency and concordance of positive samples for *NTRK* fusions using a custom nCounter assay in a real-world scenario of a single institution in Brazil. Out of 147 NSCLC patients, 12 (8.2%) cases depicted pan-NTRK positivity by IHC. Due to the absence of biological material, RNA-based NGS and/or nCounter could be performed in six of the 12 IHC-positive cases (50%). We found one case exhibiting an *NTRK1* fusion and another an *NTRK3* gene fusion by both RNA-based NGS and nCounter techniques. Both *NTRK* fusions were detected in patients diagnosed with lung adenocarcinoma, with no history of tobacco consumption. Moreover, no concomitant *EGFR*, *KRAS,* and *ALK* gene alterations were detected in *NTRK*-positive patients. The concordance rate between IHC and RNA-based NGS was 33.4%, and between immunohistochemistry and nCounter was 40%. Our findings indicate that *NTRK* fusions in Brazilian NSCLC patients are relatively rare (1.3%), and RNA-based nCounter methodology is a suitable approach for *NRTK* fusion identification in small biopsies.

## Introduction

Lung cancer remains the most deadly cancer worldwide and in Brazil^1,2^. Non-small cell lung cancer (NSCLC) is the most common histologic type of lung cancer, representing about 85% of cases. NSCLC is a heterogeneous disease, and its molecular profiling has shown the presence of molecular alterations in several oncogenes that could be therapeutically targeted, which have revolutionized the treatment of patients with NSCLC over the last years^3,4^.

The *NTRK1 (Neurotrophic Receptor Tyrosine Kinase 1)*, *NTRK2 (Neurotrophic Receptor Tyrosine Kinase 2)*, and *NTRK3 (Neurotrophic Receptor Tyrosine Kinase 3)* genes are members of the TRK (tropomyosin-receptor kinase) family, playing crucial roles in cell growth, proliferation, neuronal differentiation, survival, and metabolism in central nervous system cells^5^. The *NTRK* fusion arises as a result of genomic rearrangements (intra-chromosomal or inter-chromosomal) that juxtapose the 3′ region of *the NTRK* gene with the 5′ sequencing of the partner gene, leading to the aberrant expression of the gene and constitutive activation of the kinase domain^6^. Nevertheless, screening for *NTRK* fusions may be complex due to the diversity of both partners and breaking points locals. Larotectinib and Entrectinib are Food and Drug Administration (FDA)-approved targeted therapies that inhibit TRK fusion proteins and benefit patients with solid tumors harboring *NTRK* rearrangements^7^.

The frequency of *NTRK* fusions varies according to the tumor type, reported in 2–17% of thyroid cancers, 5–15% of salivary gland tumors, and ~ 1% of NSCLC^8–11^. Because of the low frequency and incompletely characterized partners in tumors like NSCLC, assays allowing the detection of several fusions or a two-step screening by immunohistochemistry (IHC) followed by confirmation by RNA-based next-generation sequencing (NGS) have been recommended^12–15^. However, due to the large number of driver alterations and the scarcity of tumor tissue usually available in NSCLC patients, multiplexed assays may improve *NTRK* fusion detection^16^. The nCounter assay is a robust semi-automatized platform, particularly for degraded biological material, such as formalin-fixed paraffin-embedded (FFPE) tissue, that offers a cost-effective solution with high specificity and sensitivity for detecting *NTRK* and other therapy-targeted fusions, with a reduced rate of false positive and false negative when using a custom panel with multiplex capabilities^16–19^.

Here, we aimed to evaluate the frequency of *NTRK* fusions in a real-world scenario of a routine molecular profile of NSCLC and assess the feasibility of a nCounter custom assay for rearrangement alterations in a Brazilian single center.

## Results

### Characterization of patients’ clinicopathological and molecular features

**Figure 1:**
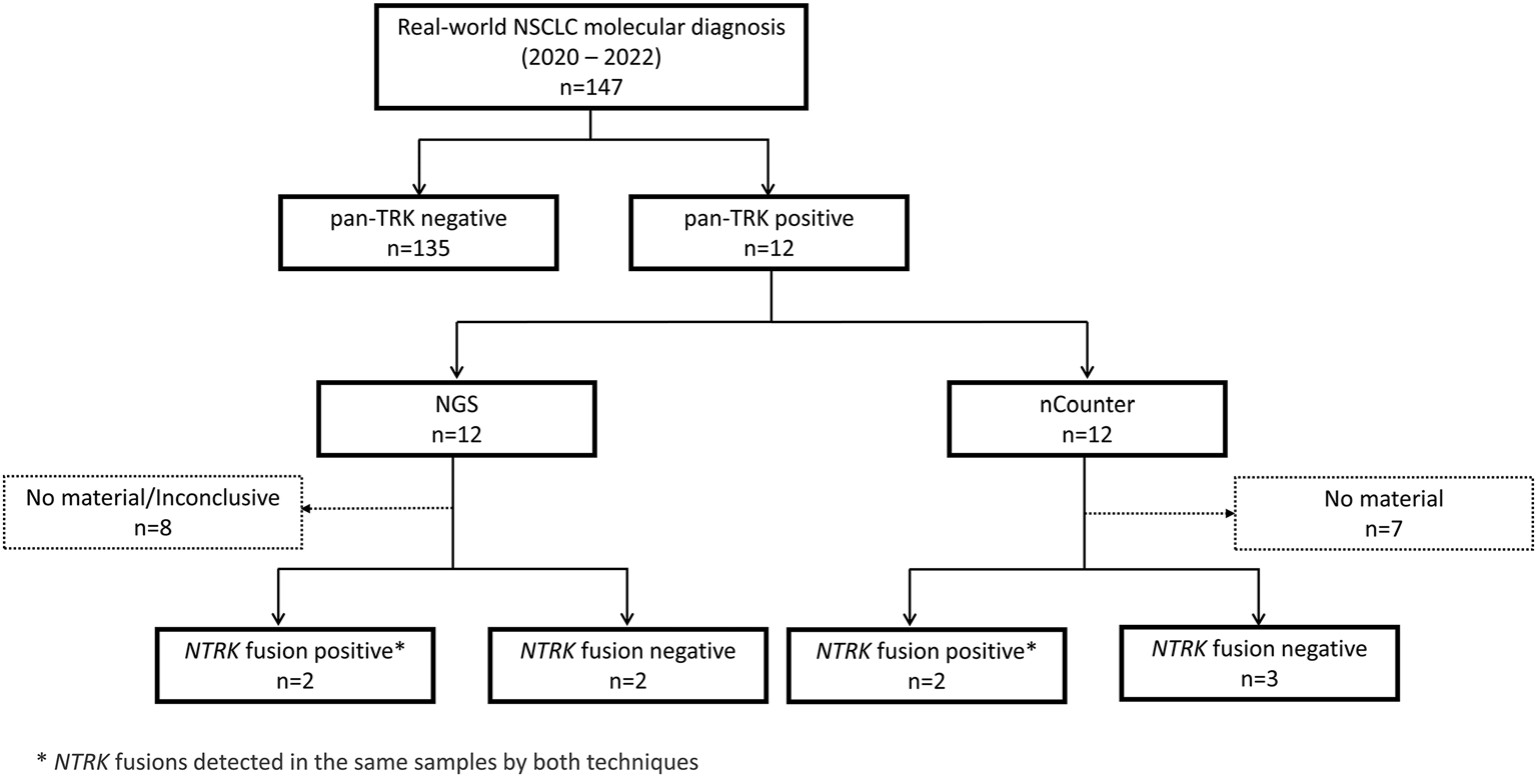
Flow chart of the study design. We selected 147 FFPE cases diagnosed with NSCLC at Barretos Cancer Hospital that were routinely evaluated for molecular diagnosis.

The clinicopathological and molecular features of the consecutive cohort of 147 formalin-fixed paraffin-embedded (FFPE) lung tumors, which were evaluated for pan-TRK, are summarized in Table 1 and Fig. 1. Molecularly, 24.5% (n = 36/147) of patients harbored *KRAS (Kirsten Rat Sarcoma Virus)* mutations, 16.3% (n = 24/147) *EGFR (Epidermal Growth Factor Receptor)* mutations, and 4.8% (n = 7/147) *ALK (Anaplastic Lymphoma Kinase)* fusions.

**Table 1:** Clinicopathological and molecular features of NSCLC consecutively evaluated for pan-TRK (n = 147).

Variable	Parameter	n	%
	Mean (min–max)	64.0 (32.0–94.0)	
Age (year)	≤ 64	77	52.4
	> 64	70	47.6
Sex	Male	83	56.5
	Female	64	43.5
Smoking	Never	37	25.2
	Quitter	49	33.3
	Current	57	38.8
	No information	4	2.7
Loss of weight^a^	No	77	52.4
	≤ 10%	23	15.6
	> 10%	29	19.8
	No information	18	12.2
ECOG PS at diagnosis	0	27	18.4
	1	79	53.7
	2	22	15.0
	3	10	6.8
	No information	9	6.1
Histology	Adenocarcinoma	109	74.1
	Squamous cell	7	4.8
	NSCLC^b^	31	21.1
Stage at diagnosis^c^	I/II	28	19.0
	III	26	17.7
	IV	83	56.5
	No information	10	6.8
Metastasis at diagnosis	No	54	36.7
	Yes, CNS	24	16.3
	Yes, Others	59	40.2
	No information	10	6.8
pan-TRK IHC	Negative	135	91.8
	Positive	12	8.2
*EGFR* mutations	Wild-type	119	81.0
	Mutated	24	16.3
	No information	4	2.7
*KRAS* mutations	Wild-type	99	67.3
	Mutated	36	24.5
	No information	12	8.2
*ALK* fusions	Wild-type	130	88.4
	Mutated	7	4.8
	No information	10	6.8
Vital status	Alive	92	62.6
	Deceased	54	36.7
	No information	1	0.7

n, number of patients; ECOG PS (Eastern Cooperative Oncology Group Performance Status); NSCLC, Non-small cell lung cancer; ^a^prior to 6 months to diagnosis; ^b^including the following histologies: NSCLC NOS (not otherwise specified), neuroendocrine large cell carcinoma, adenosquamous carcinoma, ^c^according to AJCC 8th edition; ^d^pan-TRK IHC (immunohistochemistry).

**Figure 2:**
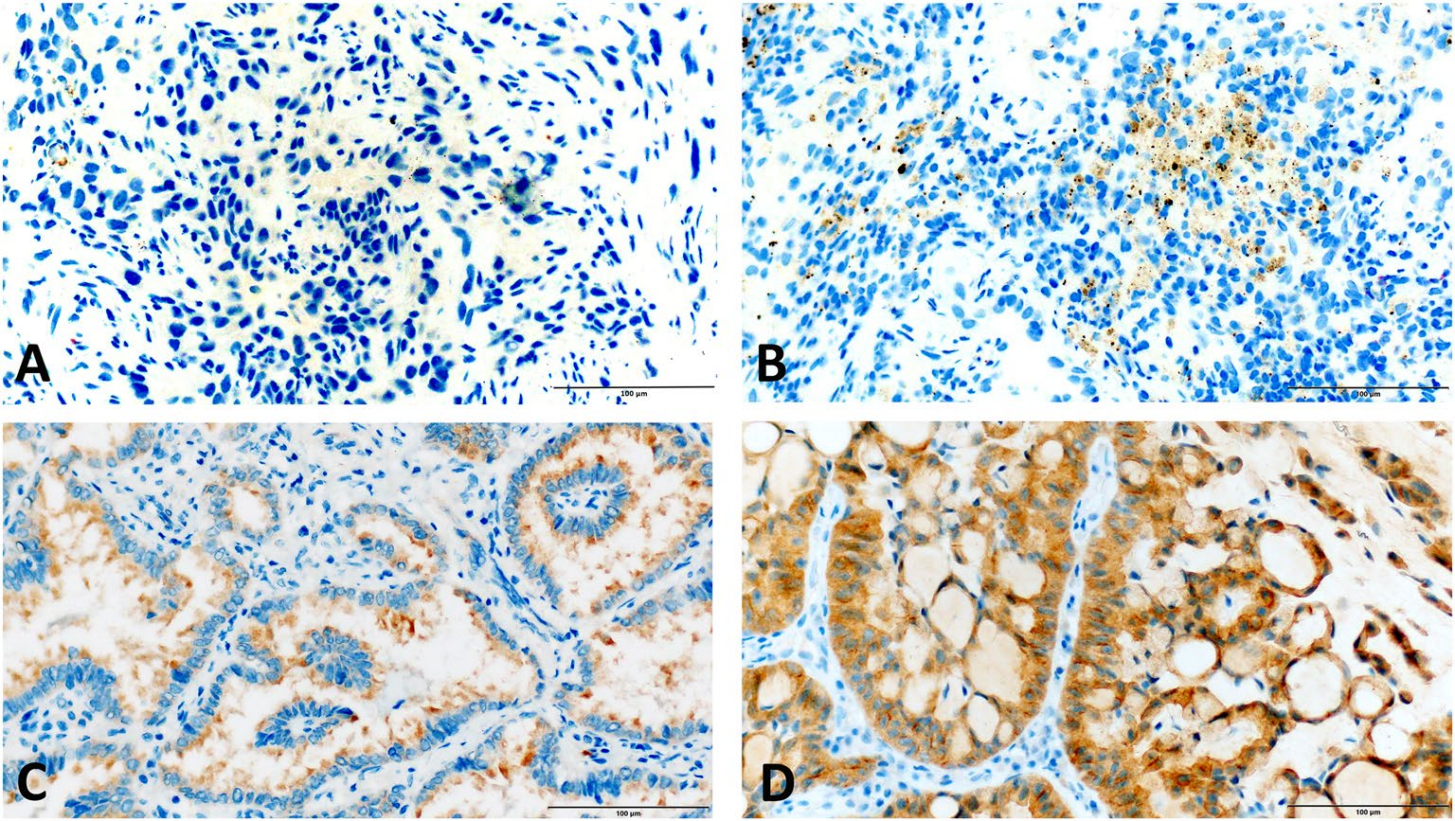
Microscopy figure of the pan-TRK immunohistochemistry. (**A**) pan-TRK-negative immunohistochemistry (**B**) pan-TRK-positive cytoplasmic 1+ (**C**) pan-TRK-positive cytoplasmic 2+ (**D**) pan-TRK-positive cytoplasmic 3+ . Brown color indicates pan-TRK positivity by DAB staining.

We observed 8.2% (n = 12/147) of cases with pan-TRK positive immunostaining (Fig. 2). The most frequent histology of IHC-positive patients was adenocarcinoma in 66.7% (n = 8/12) of patients, the median age of patients at diagnosis was 61.0 years, 58.3% (n = 7/12) were male, and 83.3% (n = 10/12) were former or current smokers (Table 2). Clinically, 58.3% (n = 7/12) of patients were diagnosed in an advanced stage of disease, 25.0% (n = 4/12) presented weight loss 6 months prior to diagnosis, and most patients presented a good performance status (Table 2). Molecularly, one patient exhibited an *EGFR* mutation p.(Leu858Arg), three patients contained the *KRAS* mutation, the p.(Gly12Cys) present in two, and a p.(Gly12Val) in one patient.

**Table 2:** Clinicopathological and molecular features of positive pan-TRK NSCLC cases (n = 12).

	Patients with positive *NTRK* (pan-TRK)															
	Age (year)	Sex	Tobacco Use	ECOG PS	Histology	Stage at diagnosis^a^	Metastasis at diagnosis	Sample specimen	Vital status	OS (months)^b^	*EGFR* mutation	*KRAS* mutation	*ALK* fusion	IHC pan-TRK	NGS^c^	nCounter^d^
ID-2068	71	Male	Never	0	LUAD	IIIB	No	Surgical	Alive	22.0	WT	WT	WT	Cytoplasmic 3+	**EML4-NTRK3**	**NTRK3**
ID-2649	54	Male	Former	2	SQC	IIIB	No	Biopsy	Alive	5.8	Missing	Missing	Inconclusive	Cytoplasmic 2+	Inconclusive	NTA
ID-2143	67	Male	Former	1	LUAD	IIIC	No	Biopsy	Deceased	4.8	WT	WT	WT	Cytoplasmic 1+	NTA	NTA
ID-2386	54	Female	Former	2	LUAD	IVB	Bone, liver, CNS	Biopsy	Deceased	1.0	WT	WT	WT	Cytoplasmic 2+	NTA	NTA
ID-2393	60	Male	Former	1	NSCLC	IVB	Bone, liver	Biopsy	Alive	6.3	WT	p.(Gly12Cys)	WT	Cytoplasmic 2+	NTA	NTA
ID-2443	74	Male	Former	2	NSCLC	IVA	Kidney	Biopsy	Alive	1.6	WT	WT	WT	Cytoplasmic 2+ and membranous 1+	NTA	Negative
ID-2406	58	Female	Current	1	LUAD	IVB	Lung/pleura, adrenal, subcutaneous	Biopsy	Deceased	3.7	WT	p.(Gly12Cys)	WT	Cytoplasmic 1+	NTA	NTA
ID-381	62	Female	Former	0	LUAD	IIIA	No	Biopsy	Alive	45.9	WT	WT	WT	Cytoplasmic 2+	Inconclusive	NTA
ID-2183	67	Male	Current	1	NSCLC	IVB	Bone, liver	Biopsy	Deceased	11.7	WT	WT	WT	Cytoplasmic 2+ and membranous 1+	Negative	NTA
ID-2642	94	Male	Former	2	LUAD	IA3	No	Biopsy	Alive	2.8	WT	p.(Gly12Val)	WT	Cytoplasmic 1+	Negative	Negative
ID-2644	59	Female	Current	1	LUAD	IVA	Lung/pleura	Biopsy	Deceased	7.5	p.(Leu858Arg)	WT	WT	Cytoplasmic 1+	NTA	Negative
ID-426	38	Female	Never	1	LUAD	IVA	Lung/pleura	Biopsy	Deceased	19.3	WT	WT	WT	Cytoplasmic 2+	**PRKAR1A-NTRK1**	**NTRK1**

Positive cases are in bold.

^a^According to AJCC 8th edition.

^b^Since the date of diagnosis.

^c^NGS (Next-generation sequencing)–Archer FusionPlex solid tumor panel.

^d^nCounter Elements XT panel. ECOG PS (Eastern Cooperative Oncology Group Performance Status), LUAD (Lung adenocarcinoma), NSCLC (Non-small cell lung cancer), SQC (Squamous Cell Carcinoma), OS (Overall Survival), IHC (Immunohistochemistry), NTA (Not Tissue Available).

### Detection of *NTRK* fusions by RNA-based NGS and RNA-based nCounter assays

Next, we tested the 12 IHC-positive cases for *NTRK* fusions using two molecular methods: NGS panel Archer FusionPlex solid tumor and our custom fusion panel nCounter Elements XT (Fig. 1). Due to the absence of biological material in the FFPE biopsies, we were able to perform the NGS test on 50.0% (n = 6/12) of the positive pan-TRK (Table 2).

**Figure 3:**
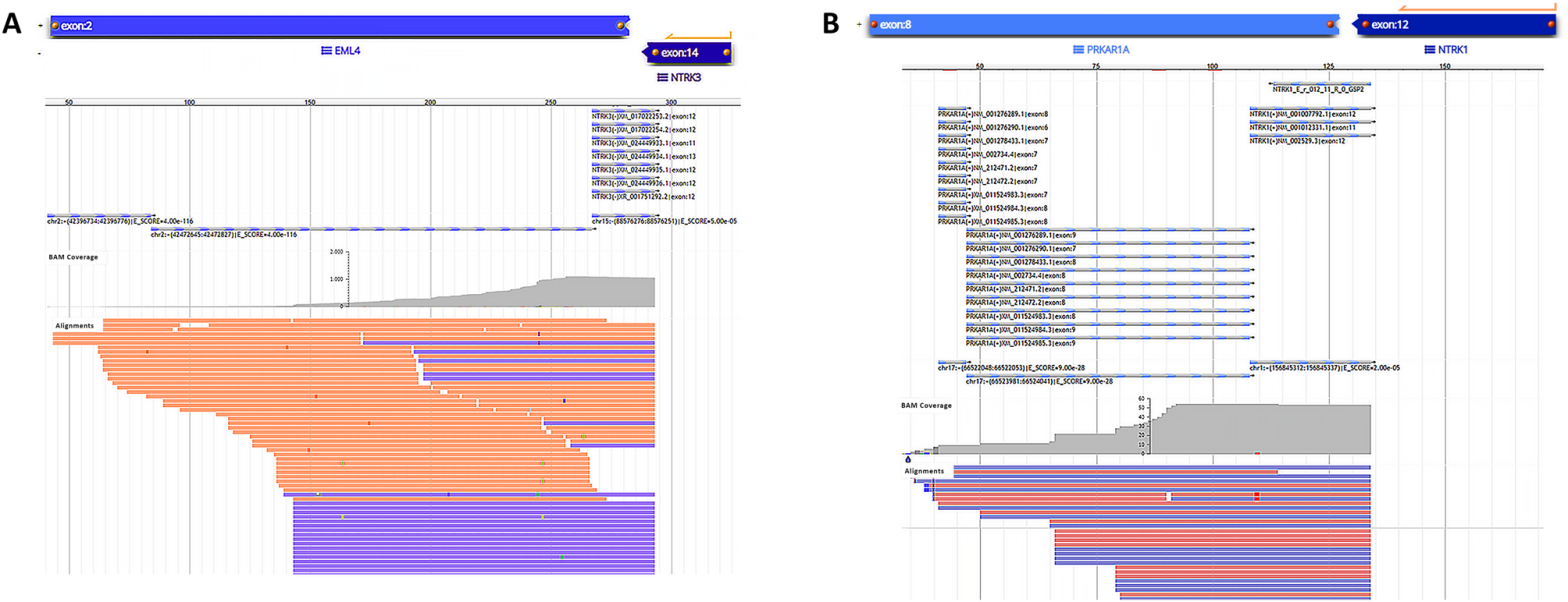
NGS analysis showing sequenced reads using Archer VR FusionPlex VR (JBrowse 1.11.6) of *NTRK* genes fusion. (**A**) Visualization of *EML4* and *NTRK3* genes. (**B**) Visualization of *PRKAR1A* and *NTRK1* genes.

**Figure 4:**
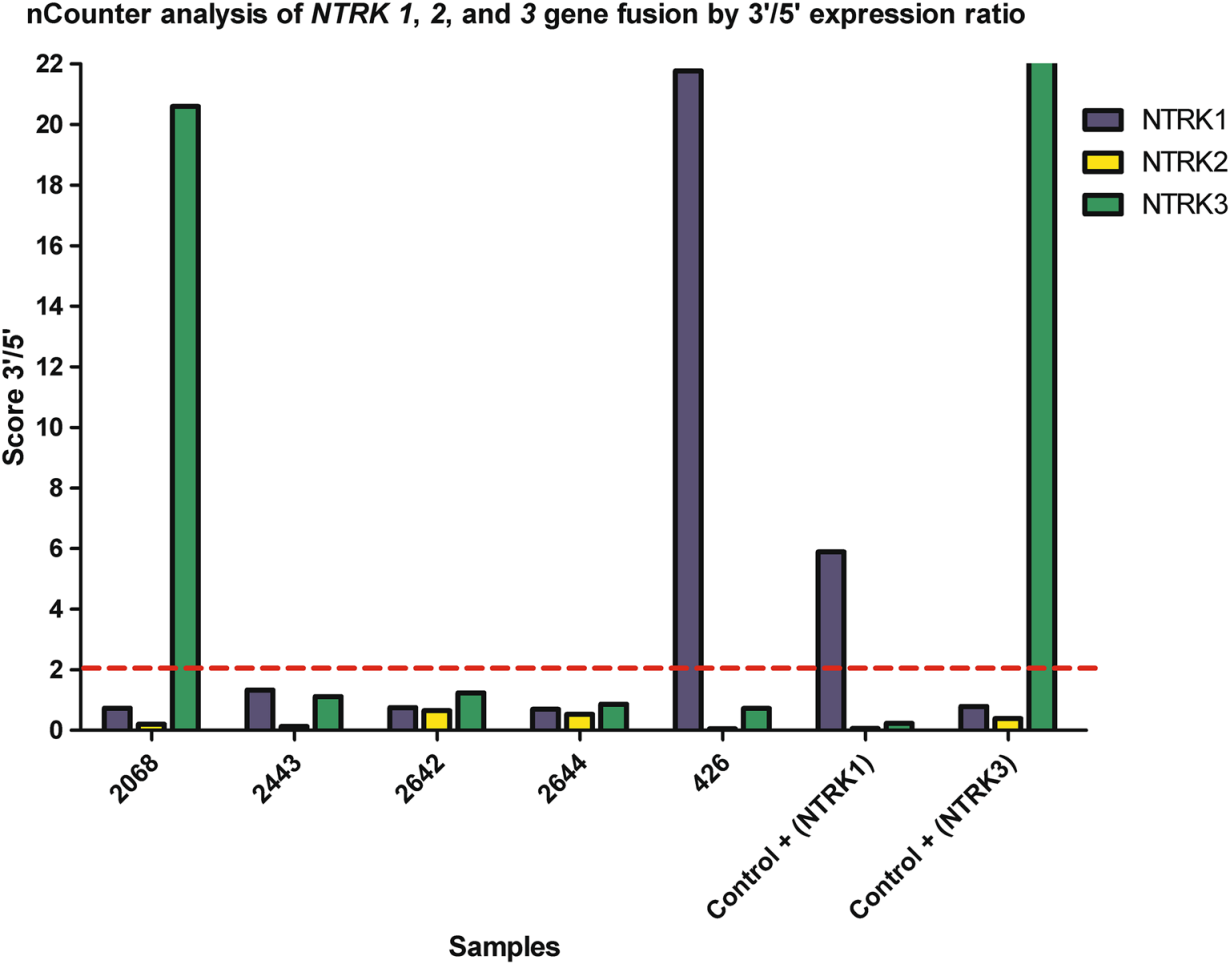
Representative graph of *NTRK* gene fusions obtained from the analyzed samples and two positive *NTRK* fusion controls (cutoff = 2). The y-axis represents the packing ratio between the 3′ and 5′ regions for the *NTRK* genes. The x-axis represents the RNA samples analyzed in the study.

Out of the six samples tested by NGS, two samples were positive for the presence of *NTRK* fusions (*EML4-Echinoderm microtubule-associated protein-like 4)-NTRK3* and (*PRKAR1A-Protein Kinase CAMP-Dependent Type I Regulatory Subunit Alpha)-NTRK1*), two were negative, and two were inconclusive (Table 2 and Fig. 3). Simultaneously, we performed our custom nCounter Elements XT fusion panel in 41.7% (n = 5/12) of the positive pan-TRK samples (Table 2). From five tested samples, two were positive for the presence of *NTRK* fusions (*NTRK1* and *NTRK3*) detected by 3′–5′ imbalance, and three samples were negative (Fig. 4). To corroborate the 3′–5′ imbalance results, we included the two positive *NTRK* fusion non-lung cancer samples in Fig. 4. Since our assay does not use specific breakpoint probes for *NTRK* genes, the fusion partners are not reported.

We further evaluated the concordance rate between the results obtained from NGS, IHC, and nCounter assays (Table 2). Three of the six samples tested using the NGS assay were also analyzed by the nCounter assay, with a concordance rate of 100% (n = 3/3; two positive and one negative samples). When comparing the results obtained from the NGS assay with the pan-TRK IHC assay, we observed a concordance rate of 33.4% (n = 2/6; two positive samples). Similarly, when comparing the nCounter assay with the pan-TRK IHC assay, we observed a concordance rate of 40% (n = 2/5; two positive samples). Additionally, when comparing only positive pan-TRK samples with IHC stain intensity defined as 2+ or 3+ with the NGS assay and nCounter assay (Table 3), we observed a concordance rate of 40.0% (n = 2/5) and 66.7% (n = 2/3), respectively.

**Table 3:** Probes of the custom NSCLC gene fusion panel of Barretos Cancer Hospital in the Elements XT nCounter.

	Elements XT nCounter panel genes and Housekeeper (HK)						
	Probes for specific genes						
	*ALK-fusion*^a^	*ROS1-fusion*^a^	*RET-fusion*^a^	*MET∆ex14*^b^	*NTRK1-fusion*	*NTRK2-fusion*	*NTRK3-fusion*
Probes 5′ and 3′ for imbalance fusion detection	*ALK_*ex1	*ROS1_*ex1_(5’_UTR)	*RET*_ex1-2		*NTRK*1_ex1	*NTRK2_*ex1	*NTRK3_*ex20b
	*ALK_*ex5	*ROS1_*ex18-19	*RET*_ex2-3		*NTRK1_*ex2-3-4	*NTRK2_*ex3	*NTRK3_*ex20
	*ALK_*ex8-9	*ROS1_*ex24	*RET*_ex6-7		*NTRK1_*ex5	*NTRK2_*ex4	*NTRK3_*ex19
	*ALK_*ex18	*ROS1_*ex29-30	*RET*_ex11		*NTRK1_*ex7	*NTRK2_*ex5-6	*NTRK3_*ex17
	*ALK_*ex22-23	*ROS1_*ex37	*RET*_ex14-15		*NTRK1_*ex14	*NTRK2_*ex8	*NTRK3_*ex9
	*ALK_*ex26-27	*ROS1_*ex40	*RET*_ex15-16-17		*NTRK1_*ex15	*NTRK2_*ex18	*NTRK3_*ex7
	*ALK_*ex29	*ROS1_*ex41/42	*RET*_ex18		*NTRK1_*ex17	*NTRK2_*ex19	*NTRK3_*ex4-5
	*ALK_*ex29_(3′_UTR)	*ROS1_*ex43_(3′_UTR)	*RET*_ex19_(3′_UTR)		*NTRK1_*ex17b	*NTRK2_*ex20	*NTRK3_*ex3
						*NTRK2_*ex21	
Probes for specific genes	*EML4_*ex13–*ALK_*ex20	*CD74*_ex6–*ROS1_*ex32	*KIFB5_*ex16–*RET*_ex12	*MET_*ex13-14			
	*EML4_*ex20–*ALK_*ex20	*SDC4*_ex2–*ROS1*_ex32	*KIFB5_*ex22–*RET*_ex12	*MET_*ex13-15			
	*EML4_*ex6–*ALK_*ex20	*SLC34A2*_ex13–*ROS1_*ex32	*KIFB5_*ex23–*RET*_ex12				
	*EML4_*ex18–*ALK_*ex20	*SLC34A2*_ex4–*ROS1_*ex32	*CCDC6_*ex1–*RET*_ex12				
	*KIF5B_*ex24–*ALK_*ex20	*EZR*_ex10–*ROS1_*ex34					
	*KIF5B_*ex17–*ALK_*ex20	*SDC4*_ex4–*ROS1_*ex34					
	*TFG_ex*5–*ALK_*ex20	*GOPC*_ex8–*ROS1_*ex35					
		*GOPC*_ex4–*ROS1_*ex36					
		*LRIG3*_ex16–*ROS1_*ex34					
Housekeeper genes (HK)		SYMPK	HPRT1	*GAPDH*	*GUSB*	*OAZ1*	*POLR2A*

^a^Previously published by Novaes et al.^16^.

^b^Previously published by Aguado et al.^35^.

Overall, the frequency of *NTRK* fusions in NSCLC patients is 1.36% (n = 2/147).

### Characterization of *NTRK*-positive patients (nCounter and NGS)

Molecularly, none of the patients had other genetic alterations in the *EGFR*, *KRAS*, and *ALK* genes (Table 2). Both male and female patients had no history of tobacco consumption, were diagnosed with lung adenocarcinoma, and presented no weight loss prior to 6 months of diagnosis (Table 2). The female patient was diagnosed at 38 with a stage IVA disease, which had metastasized to the lung and pleura, and received carboplatin with pemetrexed and pembrolizumab as first-line treatment, followed by carboplatin with paclitaxel as second-line treatment after disease progression. The other patient was male, diagnosed at 71 with a disease staged as IIIB, and was submitted to surgery (lobectomy) with adjuvant chemotherapy (cisplatin with pemetrexed) as curative treatment. None of the patients received anti-NTRK inhibitors, such as Larotrectinib or Entrectinib.

## Discussion

In the present study, we evaluated the feasibility of assessing *NTRK* fusions in a real-world scenario of routine molecular profiling of consecutive 147 NSCLC, using a custom fusion panel of nCounter assay from a single Brazilian Center.

We observed the presence of *NTRK* fusions (*NTRK1* and *NTRK3*) in 1.36% (n = 2/147) of patients. Previous studies reported that the frequency of *NTRK* fusions ranges from 0.1 to 3.3% in NSCLC patients worldwide, with fusions in *NTRK1* and *NTRK3* being more common than *NTRK2*^7,10,15,20–27^*.* In Hispanic/Latin patients with lung cancer, a recent meta-analysis reported *NTRK* fusions in 1% of patients^20^. A real-world study reported 3.5% (n = 10/289) of samples with pan-TRK expression^27^. The authors, due to insufficient material, were able to confirm the presence of *NTRK* fusion (*EML4-NTRK3*) in only one patient by NGS, rendering an *NTRK* fusion frequency of 0.35% (n = 1/289)^27^. *NTRK* fusions are reported predominantly in patients with no smoking history and diagnosed with metastatic disease^7,27^. Likewise, our patients with *NTRK* fusion were never-smokers and diagnosed with advanced disease (IVA and IIIB). Molecularly, the presence of *NTRK* fusions in our series was mutually exclusive with other driver mutations and fusions, as previously described^7,27^.

Additionally, we evaluated the concordance rate between pan-TRK immunohistochemistry, RNA-based NGS, and our custom nCounter assay. Since the majority of the cases were routine small biopsies, and a panel of IHC markers initially diagnosed the cases, then were further evaluated for molecular alterations, namely *EGFR*, *KRAS*, *ALK,* and PD-L1, no more biological material with tumor content was available for molecular validation in half of the pan-TRK-positive cases. We observed that 33.4% (n = 2/6) of tested samples using NGS were positive for *NTRK* fusion, and 40.0% (n = 2/5) of tested samples using nCounter were positive for *NTRK* fusion. We observed a concordance rate of 100% between the RNA-based NGS assay and our custom nCounter assay for *NTRK* fusion detection. Similarly, previous studies reported discordances between immunohistochemistry assays and more robust techniques (RNA-based NGS and nCounter) for *NTRK* fusion detection^15,18,28^. This may be due to methodology limitations since the pan-TRK immunohistochemistry assay detects wild-type and aberrant TRK proteins. In contrast, the RNA-based NGS and nCounter assays detect only the fusions^29^.

Importantly, detecting NSCLC patients harboring *NTRK* fusions is critical since the patients may benefit from targeted therapies, such as Larotrectinib and Entrectinib^7,11^. However, none of our patients were treated with Larotectinib or Entrectinib. Also, *NTRK* fusions are associated with resistance to EGFR-TKIs (Tyrosine Kinase Inhibitors) in NSCLC patients^7^. Thus, *NTRK* fusions have emerged as a pivotal biomarker for NSCLC patients.

Since *NTRK* fusions occur in a wide range of partners, with most of them in a low frequency, assays that identify the specific breakpoint are not ideal^11^. Our results showed high efficacy in avoiding false positive cases for *NTRK* fusions when using our custom nCounter methodology, with complete concordance with the RNA-based NGS approach. Furthermore, the nCounter technology is highly robust, with multiplex capabilities, high sensitivity, easy to execute, faster, and more cost-effective compared to NGS assays, and shows a high success rate in samples with poor quality, such as FFPE samples^19,30^. Nevertheless, one area for improvement is the absence of knowledge of the fusion partner, in addition to the high cost of the equipment. Overall, these results suggest that our custom nCounter methodology could serve as a standard approach for routine biomarker testing gene fusions (*NTRK1,2,3, ALK*, *RET (Rearranged During Transfection)*, *ROS1 (c-ros Oncogene 1),* and *MET∆ex14 (Mesenchymal Epithelial Transition exon 14 skipping*) in NSCLC patients.

These findings indicate that a custom RNA-based nCounter methodology is feasible for routine *NTRK* fusion detection and that the frequency of these alterations in Brazilian NSCLC patients is rare (1.3%).

## Methods

From 2020 to 2022, we evaluated 147 FFPE consecutive cases diagnosed with NSCLC at Barretos Cancer Hospital that were routinely evaluated for their molecular profile, which included the mutation status of *EGFR*, *KRAS*, *BRAF (V-raf Murine Sarcoma Viral Oncogene Homolog B)*, and *HER2 (Human epidermal growth factor receptor 2)* by NGS, using the TruSight Tumor 15 panel (Illumina, USA)^31,32^, immunohistochemistry (IHC) of ALK and PD-L1 (Programmed death ligand 1)^33^, and evaluation of *NTRK1/2/3* fusions. The *NTRK* fusions triage was initially done by pan-TRK IHC, followed by molecular NGS validation (Fig. 1). The clinicopathological and molecular data were collected from the patient’s medical records. The institutional review board-Barretos Cancer Hospital IRB-approved the study protocol (CAAE 05744712.3.0000.5437) and waived written informed consent due to the study’s retrospective nature.

### NTRK1/2/3 fusion detection by Immunohistochemistry

Automated immunohistochemical for TRK A, B, and C (pan-TRK) expression was performed for all cases on an automated staining system (BenchMark Ventana Ultra™) as previously described^34^. The UltraView DAB IHC detection Kit was briefly used to visualize antibody reactions. The slides were counterstained with hematoxylin, and controls were used to verify appropriate staining. To perform the reticulum staining, we used the Reticulum/Nuclear Fast Red Stain Kit (Artisan) on Artisan PRO, Dako Agilent Platform. Two pathologists reviewed the slides. We quantified the percentage of stained tumor cells in the subcellular compartments: cytoplasmic, membranous, and nuclear, as previously reported^14^. Additionally, the staining intensity for each compartment was defined on a 0 to 3 scale as follows: strong staining (3+), which was visible with the use of a 20× or 40× objective; moderate staining (2+), which required the use of a 10× or 20× objective; weak staining (1+), which involved the use of a 40× objective; and negative staining (0), which was defined as complete absence of expression (Fig. 4). As previously reported, a positive cutoff of at least 1% of tumor cells was defined^14^.

### RNA isolation

RNA isolation was performed from FFPE tumor samples, sectioned on slides with a thickness of 10μm. One slide was stained with hematoxylin and eosin (H&E) and evaluated by a pathologist for identification, sample adequacy assessment, and selection of the tumor tissue area (minimum of 60% tumor area). RNA was isolated using the RNeasy FFPE Mini Kit (Qiagen, Hilden, Germany) according to the manufacturer’s instructions. Measurement of RNA quantity was done with TapeStation 4150 (Agilent Technologies).

### Fusion detection by Archer FusionPlex solid tumor

Analysis of *NTRK* fusion was performed using the Archer FusionPlex Custom Solid Panel with Anchored Multiplex PCR (ArcherDX, Boulder, CO, USA) as previously described^34^. Briefly, the target-enriched cDNA library was prepared with the Archer FusionPlex solid tumor (ArcherDX, Boulder, CO, USA) using an amount of 100 ng of RNA as per the manufacturer’s description. In short, the reverse transcription of RNA was followed by real-time quantitative PCR (Polymerase Chain Reaction) to determine the sample quality. Then, End-repair, adenylation, and universal half-functional adapter ligation of double-stranded cDNA fragments were followed by two rounds of PCR with universal primers and gene-specific primers, covering 53 target genes that rendered the library fully functional for clonal amplification and sequencing using the MiSeq (Illumina, USA). With the Archer Analysis software version 6.0 (ArcherDX, Boulder, CO, USA), the produced libraries were analyzed for relevant fusions.

### Detection of *NTRK* fusions by nCounter Technology

Detection of *NTRK1,2,3* rearrangement was performed using the nCounter Elements XT (NanoString Technologies, Seattle, WA, USA) custom fusion panel developed at Molecular Diagnostic Laboratory, Barretos Cancer Hospital. The panel was previously designed to detect *ALK, RET,* and *ROS1*^16^ and was now updated to detect *MET*∆ex14^35^ and *NTRK1/2/3* fusions. The specific probes are detailed in Table 3.

Briefly, 100 ng RNA was hybridized with specific probes for 21 h at 67 °C. Hybridized complexes were purified using the PrepStation (NanoString Technologies, Seattle, WA, USA) and then hybridized in the cartridge. Finally, the cartridge was scanned by the Digital Analyzer (NanoString Technologies, Seattle, WA, USA) for counting transcripts. Normalization of transcripts was performed by the nSolver Analysis Software v4.0 (NanoString Technologies, Seattle, WA, USA) using the ratio of geometric mean for each sample and arithmetic mean for all samples for positive assays controls and reference gene (housekeeper). Samples with counts lower than 300 counts for the *GAPDH* gene were considered inconclusive.

Detection of *NTRK1,2,3* rearrangement was based on 3′/5′ probes imbalance, and no specific probes for breakpoints were used due to the large number of partners and breakpoints for the *NTRK* gene. The calculation of the imbalance probes was defined by the ratio between the geometric mean of 3′ probes and the average of 5′ probes, considering thresholds for positive *NTRK1/2/3* rearrangement equal to 2. Two cases were included as controls: an infant-type hemispheric glioma^34^ and an infantile fibrosarcoma harboring *NTRK1* and *NTRK3* fusions, initially detected by RNA-based NGS (Archer FusionPlex solid tumor). All analyses were performed in R environment v3.4.1.

### Statistical analysis

We described categorical variables using percentages and continuous variables using the medians for statistical analysis. To assess the concordance rate between all the techniques, we calculated the percentage of samples with concordant and discordant results between the techniques. Frequencies and medians were performed using IBM SSPS Statistics Version 25 (IBM, Armonk, Nova York, USA). Graphs were created using GraphPad Prism v5.01 (GraphPad Software Inc., Boston, Massachusetts USA).

### Statement of ethics

The institutional review board-Barretos Cancer Hospital IRB-approved the study protocol (CAAE 05744712.3.0000.5437) and waived written informed consent due to the study’s retrospective nature. All procedures were performed following the Helsinki Declaration.

## Data Availability

The data supporting this study's findings are available from Dr. Rui Manuel Reis. However, restrictions apply to the availability of patients’clinical data, which were used under ethics committee approval for the current study. Data are, however, available from the authors upon reasonable request and with permission of Dr. Rui Manuel Reis (corresponding author).
